# CMR Predictors of Favorable Outcome in Myocarditis: A Single-Center Experience

**DOI:** 10.3390/jcm13051229

**Published:** 2024-02-21

**Authors:** Anna Baritussio, Chun-Yan Cheng, Giuseppe Simeti, Honoria Ocagli, Giulia Lorenzoni, Andrea Silvio Giordani, Cristina Basso, Stefania Rizzo, Monica De Gaspari, Raffaella Motta, Giorgio De Conti, Martina Perazzolo Marra, Giuseppe Tarantini, Sabino Iliceto, Dario Gregori, Renzo Marcolongo, Alida Linda Patrizia Caforio

**Affiliations:** 1 Department of Cardiac, Thoracic, Vascular Sciences and Public Health, University of Padua and Azienda Ospedale Università Padova, 35128 Padua, Italy; anna.baritussio@aopd.veneto.it (A.B.); renzo.marcolongo@tiscali.it (R.M.); 2 Unit of Biostatistics, Epidemiology and Public Health, Department of Cardiac, Thoracic, Vascular Sciences and Public Health, University of Padua, 35131 Padua, Italy; 3 Cardiac Pathology, Department of Cardiac, Thoracic, Vascular Sciences and Public Health, University of Padua, 35128 Padua, Italy; 4 Radiology Unit, University of Padua and Azienda Ospedale Università Padova, 35128 Padua, Italy

**Keywords:** myocarditis, cardiovascular magnetic resonance, endomyocardial biopsy, outcome

## Abstract

**Background**: Cardiovascular magnetic resonance (CMR) has emerged as the most accurate, non-invasive method to support the diagnosis of clinically suspected myocarditis and as a risk-stratification tool in patients with cardiomyopathies. We aim to assess the diagnostic and prognostic role of CMR at diagnosis in patients with myocarditis. **Methods:** We enrolled consecutive single-center patients with 2013 ESC consensus-based endomyocardial biopsy (EMB)-proven or clinically suspected myocarditis undergoing CMR at diagnosis. The pre-specified outcome was defined as NYHA class > I and echocardiographic left ventricular ejection fraction (LVEF) < 50% at follow-up. **Results**: We included 207 patients (74% male, median age 36 years; 25% EMB-proven). CMR showed the highest sensitivity in myocarditis with infarct-like presentation. Patients with EMB-proven myocarditis were more likely to have diffuse LGE and right ventricular LGE (*p* < 0.001), which was also more common among patients with arrhythmic presentation (*p* = 0.001). The outcome was met in 17 patients at any follow-up time point, more commonly in those with larger biventricular volumes (*p* < 0.001), CMR-based diagnosis of dilated cardiomyopathy (*p* < 0.001), and ischemic LGE (*p* = 0.005). Higher biventricular systolic function (*p* < 0.001) and greater LGE extent (*p* = 0.033) at diagnosis had a protective effect. **Conclusions:** In our single-center cohort of rigorously defined myocarditis patients, higher biventricular systolic function and greater LGE extent on CMR at diagnosis identified patients with better functional class and higher left ventricular ejection fraction at follow-up. Conversely, larger biventricular volumes, CMR-based DCM features, and the presence of an ischemic LGE pattern at diagnosis were predictors of worse functional class and LV systolic dysfunction at follow-up. Larger prospective studies are warranted to extend our findings to multi-center cohorts.

## 1. Introduction

Myocarditis is an inflammatory disease of the myocardium with variable clinical presentation and outcome [1,2,3]. Endomyocardial biopsy (EMB) provides diagnosis and defines etiology, key to a tailored treatment [1,4,5,6]. Despite not replacing EMB, cardiovascular magnetic resonance (CMR) has become the non-invasive gold standard for diagnosing clinically suspected myocarditis [1,7,8]. CMR diagnosis was originally based on the presence of edema and/or early or late gadolinium enhancement (Lake Louise criteria [7]). These criteria have been recently updated to include parametric mapping and are expected to increase CMR diagnostic accuracy [7,8]. Several studies have addressed the prognostic role of CMR in myocarditis, but results have been conflicting [9,10,11,12,13,14,15,16].

We assessed the diagnostic and prognostic role of CMR in a single-center cohort of biopsy-proven or clinically suspected myocarditis, strictly defined following the 2013 ESC consensus criteria.

## 2. Materials and Methods

### 2.1. Study Participants

We retrospectively enrolled consecutive patients with myocarditis undergoing CMR at diagnosis during the index hospital admission. All patients in our study fulfilled the diagnosis of suspected myocarditis irrespective of CMR results. Clinically suspected (CS) myocarditis was defined according to the 2013 ESC position statement [1] as follows: ≥1 clinical presentation suggestive of myocarditis and ≥1 diagnostic criterion from different categories (ECG, increased myocytolitic enzymes, morpho-functional abnormalities at cardiac imaging, tissue characterization by CMR) in the absence of angiographically detectable coronary artery disease (CAD) and known pre-existing cardiovascular disease or extra-cardiac causes that could explain the syndrome; in asymptomatic patients ≥ 2 diagnostic tests were needed to diagnose clinically suspected myocarditis. According to the 2013 ESC consensus [1] clinical presentation of myocarditis was defined as follows: (1) infarct-like (in the absence of CAD), characterized by acute chest pain (usually starting 1–4 weeks following respiratory or gastrointestinal infection) and evidence of ST-segment elevation/depression and/or T waves inversion on ECG, with or without normal global or regional left ventricular (LV) and/or right ventricular (RV) dysfunction on echocardiography or CMR; (2) heart failure (HF), new onset or worsening (2 weeks–3 months symptoms duration, possibly started after a respiratory or gastrointestinal infection, or in the peri-partum period) or chronic HF (>3 months symptoms duration), after exclusion of CAD and other known causes of HF, with impaired systolic LV and/or RV function, with or without an increase in wall thickness, with or without dilated LV and/or RV on echocardiography or CMR, with or without increased troponin and non-specific ECG signs (bundle branch block, atrio-ventricular block, and/or ventricular arrhythmias); (3) arrhythmias, life-threatening arrhythmias and/or aborted sudden death, in the absence of CAD and known causes of HF.

Endomyocardial biopsy (EMB) was performed as clinically indicated according to current expert position papers [4,5,6] by obtaining 4–6 myocardial samples, 1–2 mm in size, from the right ventricle [4,5]; one or two frozen EMB specimens per patient were used for polymerase chain reaction (PCR) and reverse transcriptase PCR analysis and for detection of cardiotropic viruses’ genome simultaneously to histological analysis [4,17]. EMB-proven myocarditis was defined by histological (Dallas criteria), immunohistochemical (≥14 leucocytes/mm^2^ including up to 4 monocytes/mm^2^ with the presence of CD 3 positive T-lymphocytes ≥ 7 cells/mm^2^), and molecular criteria (search of cardiotropic viruses’ genome) [1].

Clinical, laboratory, and imaging data were collected at diagnosis and during follow-up. All patients were followed up at the outpatient Cardio–Immunology Clinic of the Padua University Hospital (Italy) every six months unless otherwise clinically indicated; functional status, ECG, and transthoracic echocardiography (TTE) were assessed at each visit. The pre-specified outcome was a composite of NYHA class > I and echocardiographic LV systolic dysfunction (defined as LVEF < 50%) at follow-up [6,18]. The study was approved by our Ethics Committee (protocol number 0021857); all patients provided informed consent. Patients or the public were not involved in the design, conduct, reporting, or dissemination plans of our research.

### 2.2. CMR Protocol and Analysis

Patients underwent a 1.5T CMR scan including long and short-axis cine sequences, T2-weighted (T2w), early gadolinium enhancement (EGE), and late gadolinium enhancement (LGE) sequences. EGE and LGE images were respectively acquired 1–3 min and 10–15 min after intravenous injection of 0.2 mmol/kg of gadolinium-based contrast agent (GBCA), in identical planes to cine images.

Biventricular volumes and function were assessed with dedicated software (Circle CVI42^®^, Calgary, AB, Canada, version 5.11), and all measurements were indexed to body surface area. Myocardial edema on T2w images was defined by a ratio of signal intensity (SI) between the myocardium and the mean SI of the skeletal muscle ≥ 2 [7]; in order to obtain the T2 SI ratio, we first outlined left ventricular endocardial and epicardial contours on each short axis T2w slice and then we drew the contour for a region of interest (ROI) in a large area of the skeletal muscle closest to the heart and to the center of the reception field of the coil (usually in the M. serratus anterior). LGE was defined as subendocardial or transmural (ischemic pattern) if involving <50% or ≥50 of wall thickness, respectively, and mid-wall/epicardial (non-ischemic pattern). LGE distribution was defined as diffuse when involving multiple territories. LGE extent was quantified, blinded to EMB results, using the full width at half maximum method by dedicated software (Circle CVI 42^®^, Calgary, AB, Canada, version 5.11) [19]. As CMR scans were performed over the last decade, for data uniformity, CMR findings were considered consistent with myocarditis if at least 2 of the following criteria were present: (a) regional or global myocardial signal intensity (SI) increase on T2w images, (b) increased global myocardial EGE ratio between myocardium and skeletal muscle in gadolinium-enhanced sequences, (c) at least one focal lesion with non-ischemic regional distribution on LGE sequences [7]. Analysis was carried out according to recommendations of the Society for Cardiovascular Magnetic Resonance [20].

### 2.3. Statistical Analysis

Descriptive variables were reported as mean ± SD or as median (IQR); categorical variables were reported as absolute numbers and percentages. The Kruskal–Wallis test and Pearson chi-square tests were used to evaluate differences in distribution according to myocarditis diagnosis (EMB vs. CS) and to clinical presentation, respectively, for continuous and categorical variables. The Spearman correlation was used to test the correlation between echocardiographic LVEF and CMR LVEF. Given the low number of events at follow-up, only univariate analysis was performed to assess the effect of potential outcome predictors using Cox Proportional Hazard models. Results were reported as Hazard Ratio (HR), 95% Confidence Interval (CI), and *p*-value. Outcome predictors were assessed at pre-defined follow-up time points (0–6, 6–12, 12–24 > 24 months, and at any time point). A *p*-value < 0.05 was considered significant. The analyses were performed using R software (version 4.0.2) with the packages rms and survival [21,22,23,24].

## 3. Results

### 3.1. Baseline Patient Characteristics

We retrospectively included 207 patients, 74% male, median age 36 (24–47) years, 156 (75%) with CS, and 51 (25%) with EMB-proven myocarditis (Table 1).

Of the overall cohort, 63% of patients reported symptoms before diagnosis; 47% had chest pain. The majority of patients had an acute presentation, as shown by the median symptoms’ duration in the overall cohort. Anti-heart auto-antibodies (AHA), tested in 182 patients, were positive in 107 (59%), the most prevalent being organ-specific AHA (92%). All patients received standard care. Previous respiratory or gastrointestinal viral infection (1–4 weeks prior to symptoms’ onset) was more frequent, and troponin I was higher in patients with CS myocarditis.

Patients with EMB-proven myocarditis were mainly older females with immune diseases, longer symptom duration, more advanced NYHA, arrhythmias, signs of left and right HF, and lower ventricular function on TTE. Histological types of EMB-proven myocarditis were lymphocytic (n = 48), giant-cell (n = 2), and polymorphic (n = 1); six patients (3%) had a fulminant presentation. Viral genomes were detected in eight biopsy specimens: Parvovirus B19 (PVB19) (n = 5), Epstein–Barr virus (n = 1), Cytomegalovirus (n = 1), and Influenza A virus (n = 1). All patients received standard optimal medical therapy (OMT) according to international guidelines; a third of patients were treated with beta-blockers (more frequently biopsy-proven patients), 40% were treated with renin–angiotensin–aldosterone system inhibitors (more frequently biopsy-proven patients), and a few patients received anti-arrhythmic treatment, mainly amiodarone. Twenty-two EMB-proven virus-negative patients (11%) started immunosuppressive therapy on top of standard OMT, 12 for worsening/unremitting HF; LVEF improved in all but one.

### 3.2. CMR Findings—Overall Cohort

CMR findings are summarized in Table 2. 

In the overall cohort, biventricular volumes and function were preserved on CMR, and there was a positive correlation between LVEF on CMR and on TTE at diagnosis (r = 0.65, *p* < 0.001). Myocardial edema was found in more than half of patients, affecting more than one LV wall (57%), the lateral wall (29%), the anterior wall (1%), the septum 6%, and the inferior wall (5%). Diffuse edema was present in 2% of cases. 

Nearly all patients showed LGE on post-contrast images, with a non-ischemic pattern in 93% of cases. LGE was more commonly found in more than one site (69%) and in the lateral wall (22%), while the remaining walls were less commonly affected (inferior wall 3%, septum 5%, diffuse distribution 1%). Pericardial and pleural effusion were found in 48 (23%) and 37 (18%) patients. 

The CMR-based diagnosis was consistent with myocarditis in 78% of patients, dilated cardiomyopathy (DCM) in 11%, structurally normal heart in 3%, ischemic necrosis in 1%, and another final diagnosis in 15 (7%). CMR showed the highest sensitivity (CMR features of myocarditis among EMB-proven myocarditis cases) in diagnosing myocarditis with infarct-like presentation (100%) but lower sensitivity in those with arrhythmias (50%) and heart failure (28%). Only three EMB-proven patients were asymptomatic; CMR did not show myocarditis features in any of them.

Patients presenting with HF (as compared to the remaining population with other types of clinical presentation) had larger LV volumes (LVEDV 132 ± 54 mL/m^2^ vs. 89 ± 18 mL/m^2^, *p* < 0.001; LVESV 92 ± 57 mL/m^2^ vs. 38 ± 14 mL/m^2^, *p* < 0.001) and lower biventricular systolic function (LVEF 35 ± 16% vs. 58 ± 8%, *p* < 0.001; RVEF 46 ± 14% vs. 59 ± 7, *p* < 0.001). They more likely showed a diffuse myocardial edema pattern (7% vs. 2%, *p* = 0.022). LGE was more likely to be absent in patients with HF presentation (LGE absence 17% vs. 5%, *p* = 0.007) and affected fewer myocardial segments (2.7 ± 1.8 vs. 4.1 ± 3.0, *p* = 0.014); LGE had a lower extent in HF presentation (3.6 ± 3.9 g vs. 5.9 ± 5.9 g, *p* = 0.029) and more commonly a diffuse distribution (3% vs. 1%, *p* = 0.046). Patients with arrhythmic presentation more commonly showed right ventricular LGE (12% vs. 1%, *p* = 0.001).

### 3.3. CMR Findings—CS vs. EMB-Proven Cohort

The median time between EMB and CMR was 5 days (2.25–6). A CMR diagnosis of myocarditis was more common among patients with CS myocarditis (87% vs. 47%), while a CMR diagnosis of DCM was more common among EMB-proven myocarditis patients (42% vs. 1%, *p* < 0.001).

**Figure 1:**
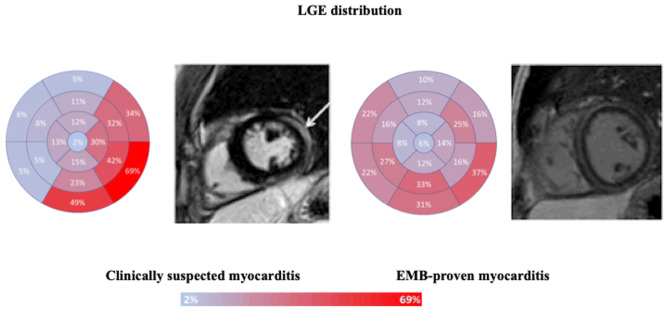
LGE distribution, according to the American Heart Association bull’s eye plot, in patients with clinically suspected or EMB-proven myocarditis. Focal LGE distribution, mainly located in inferior and lateral walls in clinically suspected myocarditis, as shown by the short axis mid-cavity post-contrast sequence displaying epicardial LGE of the lateral wall (**left,** white arrow). Diffuse LGE distribution in EMB-proven myocarditis, with evidence of circumferential mid-wall/subepicardial LGE (**right**).

CS myocarditis more commonly showed myocardial edema in the inferior (34% vs. 14%, *p* = 0.006) and lateral walls (38% vs. 14%, *p* = 0.001). Despite no difference in LGE prevalence and extent between the two groups, CS myocarditis more frequently had LGE in the lateral (66% vs. 45%, *p* = 0.008) and inferior walls (61% vs. 41%, *p* = 0.014), while EMB-proven myocarditis had a more diffuse LGE distribution and a higher prevalence of right ventricular LGE (8% vs. 0, *p* < 0.001) (Figure 1).

### 3.4. Prognosis in the Overall Cohort

Follow-up data were available for 201 patients with a median follow-up of 32 months (IQR 14–61). At the last follow-up, patients with EMB-proven myocarditis, compared to those with CS myocarditis, had worse clinical status and lower LVEF on TTE and were more likely to be on HF and/or anti-arrhythmic therapy (Table 3).

One EMB-proven patient underwent a heart transplant, but no patient died. The pre-specified composite outcome was met in 17 patients. As shown in Table 4, across each time point at follow-up, larger biventricular volumes and diffuse wall motion abnormalities at diagnosis increased the risk of the end-point, while higher baseline biventricular systolic function was associated with favorable outcomes. 

While edema and EGE at baseline were never associated with the end-point, transmural LGE was associated with the end-point at 6–12 months follow-up. Pericardial and pleural effusion were associated with the end-point at each time point, while an epicardial stria of LGE showed a protective effect both at 6–12 months (HR 0.04, 95% CI 0–0.68, *p* = 0.026) and at “any time point” (HR 0.07, 95% CI 0.01–0.53, *p* = 0.01). CMR diagnoses of DCM and of ischemic LGE were strongly associated with the end-point (0–6 months: DCM HR 14.8, 95% CI 1.81–122, *p* = 0.012, ischemic LGE HR 51.8, 95% CI 3.05–878, *p* = 0.006; at any time point: DCM HR 40.7, 95% CI 5.31–312, *p* < 0.001, ischemic LGE HR 56.4, 95% CI 3.41–932, *p* = 0.005). 

## 4. Discussion

### 4.1. Diagnostic Role of CMR in Suspected Myocarditis 

CMR confirmed the clinical suspicion of myocarditis in 78% of our overall cohort, but in 53% of the biopsy-proven patients, CMR failed to confirm the diagnosis, mainly reporting DCM features, as previously described [25]. In keeping with previous findings from Francone et al. [25], in our cohort, CMR showed the highest sensitivity in patients with infarct-like presentation. This may reflect a higher accuracy of the old Lake Louise CMR criteria in detecting focal myocardial disease, typical of infarct-like presentation, as compared to diffuse myocardial processes, more common in myocarditis presenting as HF [8]. Indeed, in our cohort, EMB-proven myocarditis patients with HF were more likely to have diffuse edema and LGE, as compared to patients with an infarct-like presentation, who mainly showed edema and LGE of the inferior and lateral LV walls. Moreover, the frequency of edema was lower in our EMB-proven patients, suggesting a possible underestimation of this feature by standard T2-weighted sequences. To overcome some of these limitations, the revised Lake Louise criteria now include parametric mapping that does not rely on the presence of remote, normal myocardium [8] and is ideal for detecting diffusely diseased myocardium. Preliminary data are promising in this regard [26]; in a cohort of 102 patients with suspected myocarditis, of whom 30 were biopsy-proven, the addition of parametric mapping increased the diagnostic accuracy of CMR in patients with heart failure while showing no significant diagnostic improvement in those with infarct-like or arrhythmic presentation. Interestingly, this was also confirmed when considering only patients with a biopsy-proven diagnosis.

### 4.2. CMR Features in EMB-Proven versus CS Myocarditis

In our study, EMB-proven myocarditis showed more advanced reverse remodeling and lower biventricular systolic function, likely reflecting the more frequent use of EMB in most severe presentations [2,5,6,27]. Although we confirmed in the overall cohort a high prevalence of edema and LGE involving three LV myocardial segments [9,15,16], we found no difference in LGE frequency and extent between CS and EMB-proven groups. 

However, LGE was less frequent among our EMB-proven patients with HF, and its extent was lower as compared to those without HF presentation. This is in keeping with a previous study on CS myocarditis describing higher LGE prevalence and a higher extent among patients with infarct-like as compared to those without infarct-like presentation [13]. In our cohort, right ventricular LGE was only found in EMB-proven myocarditis less frequently than previously reported [28], possibly due to the higher prevalence of CS myocarditis with an infarct-like presentation in our cohort. Our patients with an arrhythmic presentation more commonly had RV LGE on CMR, in keeping with the known arrhythmogenicity of right ventricular scars in different non-ischemic cardiomyopathies [29,30]. 

### 4.3. Prognostic Role of CMR in Myocarditis 

The main prognostic implication of our study is that in the overall cohort, higher biventricular systolic function and greater LGE extent on CMR at diagnosis predict more favorable outcomes in myocarditis. Conversely, larger biventricular volumes, CMR-based DCM features, and the presence of an ischemic LGE pattern at diagnosis were predictors of worse outcomes. The very low rate of major adverse cardiovascular events (MACE) in our cohort (only one patient underwent a heart transplant and no one died) might relate to the relatively preserved LVEF at baseline in the CS cohort, to a potential selection bias as the more severely ill patients, known to have a worse outcome, may not receive CMR and to the favorable effect of immunosuppression in the biopsy-proven group by improving patients’ clinical status and left ventricular function. Indeed, left and/or right ventricular dysfunction at diagnosis is known to be the main predictor of dismal prognosis in myocarditis [1,2,3,11,17,31,32,33,34] and of lower LVEF at follow-up, in keeping with our findings. In addition, autoimmune etiopathogenesis was associated with worse outcomes in the pre-immunosuppression era [35], and immunosuppression has a beneficial effect on LVEF and life-threatening arrhythmia, both surrogate end-points of outcome [36,37,38,39]. 

The most novel finding of our study was that the extent of myocardial LGE on CMR at diagnosis predicted more favorable outcomes when assessed at any time point of follow-up. It is well known that LGE represents expanded interstitial space, rather than peremptorily indicating irreversibly damaged myocardium [8], and, especially in the acute setting, it may also represent myocardial edema, as evidenced by complete LGE resolution on follow-up CMR in some myocarditis cases [40]. The protective role of LGE in our cohort may, therefore, reflect the presence or co-existence of myocardial edema, a marker of a reversible process, thus explaining the better outcome, leading to higher LVEF and better functional status at follow-up. Edema did not correlate with outcome in our cohort, but this may be due to the known lower sensitivity of T2w sequences to detect edema, as compared to other sequences (i.e., T2 mapping). Moreover, the non-ischemic distribution of LGE typical of myocarditis patients, which spares the sub-endocardium, may as well explain why our myocarditis patients with non-ischemic LGE at baseline CMR showed higher LVEF at follow-up, as opposed to the negative predictive role of ischemic LGE on follow-up LVEF in our cohort, in keeping with previous studies [13]. It has to be reminded, among the possible explanations, that in the absence of EMB confirmation, an infarct-like LGE pattern, rather than reflecting myocarditis, may represent myocardial infarction with non-obstructive coronary arteries, a clinical entity known to have a worse outcome. The prognostic role of LGE in myocarditis is presently unclear. Two recent meta-analyses on more than 2000 patients with CS myocarditis reported the association of LGE and reduced LVEF with worse outcomes [41,42], although it is unclear whether LGE had an independent prognostic role over LVEF. Similarly, a study on 670 patients with suspected myocarditis and mildly impaired LVEF (50%) found that LGE, especially septal and mid-wall, doubled the risk of MACE [12]; however, among patients with LVEF < 40% (who had a worse outcome as compared to those with higher LVEF), LGE presence did not provide additional prognostic stratification. Therefore, the univocal, independent prognostic role of LGE over LVEF is unclear. The ITAMY study also found that anteroseptal LGE predicted worse outcomes in infarct-like CS myocarditis with normal LVEF [9]. On the other hand, in keeping with our findings, other studies have found that LGE does not add prognostic value to reduced baseline LVEF [10,16], although end-points were different. A recent study has also shown low long-term sudden cardiac death risk in patients with non-ischemic LGE and normal LV volumes and LVEF, with mortality mainly driven by age and not by LGE presence, location, or extent [43]. What seems to emerge from available research studies is that the prognostic capability of imaging features in myocarditis may rather derive from their incremental value when considered together than considering each of them separately; in a recent study on 455 patients with myocarditis (defined according to ESC criteria [1]), the addition of imaging markers, such as LVEF, LGE (when considering both its extent and location, i.e., mid-wall distribution), and left ventricular strain, to clinical characteristics and symptoms improved the overall prognostic accuracy [44]. It may be supposed that the heterogeneous nature of myocarditis may also account for heterogeneity in its risk predictors.

As previously reported, in our cohort, myocardial edema on CMR at diagnosis did not show a prognostic association with the outcome, in keeping with previous studies [45,46], and this may be due to the reversible myocardial injury that edema represents. Other studies have instead shown the negative prognostic role of persisting edema on follow-up CMR [40], likely as an expression of ongoing myocardial inflammation and, therefore, of an active disease process. The advent of parametric mapping has shown the incremental prognostic role of T2 mapping in risk stratification, as compared to standard T2-weighted sequences that are subject to artifact and may therefore be less sensitive [45]. Moreover, both T1 and T2 mapping appeared promising as tools to monitor the inflammatory state [46]. 

### 4.4. Limitations

We acknowledge that EMB was performed as clinically indicated, according to current expert position papers [4,5,6], only in a minority of patients; however, the remaining clinically suspected myocarditis cases were strictly defined following the 2013 ESC consensus criteria [1]. We, therefore, believe that our cohort represents a real-life description of myocarditis patients. CMR was performed at the clinician’s discretion in hemodynamically stable patients, potentially introducing a selection bias, as patients with worse presentations are less likely to receive CMR. The higher prevalence of infarct-like CS myocarditis, with a more favorable disease course, the absence of hard MACE at follow-up, and the small number of patients meeting the surrogate end-point may have blunted the additional predictive role of CMR features. Moreover, the EMB-proven group had more diffuse myocardial involvement, which is less accurately detected by standard CMR tissue characterization sequences, and parametric mapping sequences were not systematically acquired in our cohort. Although the prognostic value of parametric mapping sequences has been less explored, T2 mapping has been shown to have a role in predicting MACE and HF hospitalization in patients with myocarditis [45], as long as an extracellular volume that, when increased, portends a worse prognosis, irrespective of the presence of LGE [47]; further studies are therefore needed to clarify the role of parametric mapping as a risk stratification tool. We used the FWHM method to quantify LGE extent, and we cannot exclude that results may have been different by using alternative LGE quantification methods (i.e., n-Standard Deviation techniques); however, the FWHM method has shown to be the optimal semi-automated quantification method in risk-stratifying patients with suspected myocarditis, showing the strongest association with MACE and the highest technical consistency [19]. Finally, a follow-up CMR could have provided further insights into its prognostic role [40]. 

## 5. Conclusions

In our single-center cohort of rigorously defined myocarditis patients, higher biventricular systolic function and greater LGE extent on CMR at diagnosis identified patients with better functional class and higher left ventricular ejection fraction at follow-up. Conversely, larger biventricular volumes, CMR-based DCM features, and the presence of an ischemic LGE pattern at diagnosis were predictors of worse functional class and LV systolic dysfunction at follow-up. Larger prospective studies are warranted to extend our findings to multi-center cohorts. 

## Figures and Tables

**Table 1:** Patients’ characteristics at diagnosis.

	All n = 207	EMB-Proven Myocarditis n = 51	Clinically Suspected Myocarditis n = 156	*p*-Value
Age, years	36 (24–47)	41 ± 15	35 ± 14	0.01
Female	54 (26%)	20 (39%)	34 (22%)	0.014
FHx ID *	23 (11%)	7 (14%)	16 (10%)	0.52
FHx CAD †	42 (21%)	12 (25%)	30 (20%)	0.45
Previous viral infection *	91 (45%)	12 (24%)	79 (52%)	<0.001
ID	25 (12%)	11 (22%)	14 (9%)	0.017
Arrhythmic presentation	16 (8%)	8 (16%)	8 (5%)	0.014
HF presentation	41 (20%)	32 (63%)	9 (6%)	<0.001
Infarct-like presentation	144 (70%)	9 (18%)	135 (87%)	<0.001
Symptom duration before diagnosis (mo)	1.00 (0.17–6.00)	2.00 (1.00–11.25)	0.15 (0.03–1.25)	<0.001
NYHA class at diagnosis ‡, I	167 (81%)	18 (36%)	149 (96%)	<0.001
II–IV	39 (19%)	32 (64%)	7 (4%)	
Left HF	37 (18%)	31 (61%)	6 (4%)	<0.001
Right HF	14 (7%)	12 (24%)	2 (1%)	<0.001
Sinus rhythm *	198 (97%)	47 (92%)	151 (99%)	0.057
Atrial fibrillation *	3 (1%)	2 (4%)	1 (1%)	
Left bundle branch block §	9 (4%)	8 (16%)	1 (1%)	<0.001
Right bundle branch block §	13 (6%)	6 (12%)	7 (5%)	<0.001
Troponin I peak, ng/L ?	3350 (276–10,575)	210 (0–7930)	4963 (1100–11,100)	<0.001
CRP peak, mg/dL #	19.2 (4.9–50.0)	6.8 (3.1–21.8)	25.5 (7.0–51.5)	0.004
LV diastolic diameter, mm **	51 ± 7	56.2 ± 9.8	49.6 ± 4.9	<0.001
Left atrial volume mL/m^2^ ‡‡	33 ± 13	43 ± 18	30 ± 10	<0.001
LVEDVi, mL/m^2^ ?	71 ± 25	92 ± 39	64 ± 12	<0.001
LVEF, % §§	52 ± 14	36.9 ± 16.2	57.0 ± 7.3	<0.001
RVEDA, cm^2^ ??	21.3 ± 5.2	23.0 ± 7.3	20.8 ± 4.1	0.21
RVFAC, % ##	42 ± 10	33.6 ± 10.2	45.4 ± 7.1	<0.001

Data are n (%), mean ± SD, or median (IQR). FHx, family history; ID, immune-mediated disease; CAD, coronary artery disease; HF, heart failure; NYHA, New York Heart Association; CRP, C-reactive protein; LVEDV, left ventricular end-diastolic volume; LVEF, left ventricular ejection fraction; RVEDA, right ventricular end-diastolic area; RVFAC, right ventricular fractional area change. Data available in * 204, † 199, ‡ 206, § 202, ? 178, # 173, ** 154, ‡‡ 144, §§ 191, ?? 139, ## 133 patients.

**Table 2:** CMR findings.

	All n = 207	EMB-Proven Myocarditis n = 51	Clinically Suspected Myocarditis n = 156	*p*-Value
LVEDVi mL/m^2^	90 (79–106)	110 (92–156)	86 (78–98)	<0.001
LVESVi mL/m^2^	38 (31–48)	69 (41–118)	36 (30–42)	<0.001
LVSV mL	93 (76–106)	67 (53–86)	96 (83–111)	<0.001
LVEF %	57 (51–62)	33 (23–51)	59 (55–62)	<0.001
LV mass, g/m^2^	61 (52–74)	76 (58–85)	59 (51–69)	<0.001
LV regional WMA	61 (29%)	21 (41%)	40 (26%)	0.035
LV diffuse WMA	33 (16%)	28 (55%)	5 (3%)	<0.001
RVEDVi mL/m^2^	83 (74–96)	84 (70–100)	83 (74–94)	0.86
RVESVi mL/m^2^	35 (28–44)	45 (31–57)	34 (27–40)	<0.001
RVSV mL	90 (72–106)	68 (56–86)	96 (81–109)	<0.001
RVEF %	58 (53–63)	47 (36–56)	59 (56–60)	<0.001
Edema	129 (62%)	19 (37%)	110 (70%)	<0.001
LV segments with edema	3 (2–5)	2 (2–6)	3 (2–4)	0.53
Transmural edema	48 (38%)	7 (37%)	41 (38%)	0.9
EGE	83 (40%)	13 (25%)	70 (45%)	0.015
LGE	193 (93%)	45 (88%)	147 (94%)	0.15
LV segments with LGE	3 (2–5)	2 (2–4)	3 (2–5)	0.24
Transmural LGE	41 (21%)	14 (30%)	27 (18%)	0.081
LGE mass, g	3.5 (1.7–7.5)	3.3 (1.6–7.6)	3.5 (1.7–7.5)	0.88
LGE mass, % of LV	3.0 (1.5–6.1)	2.5 (1.0–6.2)	3.2 (1.6–5.9)	0.29

Data are n (%), mean ± SD, and median (IQR). LVEDVi, indexed left ventricular end-diastolic volume; LVESVi, indexed left ventricular end-systolic volume; LVSV, left ventricular stroke volume; LVEF, left ventricular ejection fraction; WMA, wall motion abnormality; RVEDVi, indexed right ventricular end-diastolic volume; RVESVi, indexed right ventricular end-systolic volume; RVSV, right ventricular stroke volume; EGE, early gadolinium enhancement; LGE, late gadolinium enhancement.

**Table 3:** Patients’ characteristics at the last follow-up.

	All n = 201	EMB-Proven Myocarditis n = 49	Clinically Suspected Myocarditis n = 152	*p*-Value
Duration of the follow-up (months)	32 (14–61)	42 (17–64)	30 (14–57)	0.16
Dead or transplanted	1 (0%)	1 (2%)	0 (0%)	0.074
NYHA class, I	188 (94%)	40 (85%)	148 (97%)	0.001
II–IV	11 (6%)	7 (15%)	4 (3%)	
Sinus rhythm	187 (96%)	40 (87%)	147 (99%)	<0.001
Atrial fibrillation	4 (2%)	4 (9%)	0 (0%)	
Beta-blockers	63 (31%)	33 (67%)	30 (20%)	0.002
Ivabradine	6 (3%)	4 (8%)	2 (1%)	0.014
ACE inhibitors *	59 (32%)	20 (44%)	39 (27%)	0.033
ARB †	10 (9%)	8 (22%)	2 (3%)	<0.001
Amiodarone	2 (1%)	2 (4%)	0 (0%)	0.012
Anticoagulants *	7 (4%)	7 (16%)	0 (0%)	<0.001
LV end-diastolic diameter, mm ‡	49.4 ± 6.3	52.1 ± 8.8	48.6 ± 5.1	0.005
Left atrial volume, mL/mq §	23.3 ± 10.1	26.0 ± 13.5	22.0 ± 7.6	0.32
LVEDVi, mL/mq ?	59 ± 17	69 ± 28	56 ± 11	<0.001
LVEF, % #	62.4 ± 8.1	57.4 ± 11.3	64.0 ± 6.0	<0.001
RVEDA, cm ‡	18.5 ± 4.6	18.8 ± 6.5	18.5 ± 3.9	0.50
RVFAC, % **	48.9 ± 8.0	48.3 ± 10.1	49.2 ± 7.2	0.68

Data are n (%), mean ± SD, or median (IQR). ACE, angiotensin-converting enzyme; ARB, angiotensin receptor blockers; for other abbreviations see Table 1. Data available in * 187, † 110, ‡ 189, § 108, ? 195, # 198, ** 188 patients.

**Table 4:** Univariate Cox-predictors of the surrogate end-point at different follow-up visits.

	0–6 Months	6–12 Months	12–24 Months	>24 Months	Any Time Point
Number of Events	10	9	9	3	17
LVEDVi mL/m^2^	HR 1.01 (95% CI 1.01–1.02, *p* < 0.001)	HR 1.02 (95% CI 1.01–1.03, *p* < 0.001)	HR 1.02 (95% CI 1.01–1.03, *p* < 0.001)	HR 1.05 (95% CI 1.01–1.08, *p* = 0.006)	HR 1.02 (95% CI 1.02–1.03, *p* < 0.001)
LVESVi mL/m^2^	HR 1.01 (95%CI 1.01–1.02, *p* < 0.001)	HR 1.01 (95% CI 1.01–1.02, *p* < 0.001)	HR 1.02 (95% CI 1.01–1.03, *p* < 0.001)	HR 1.06 (95% CI 1.01–1.12, *p* = 0.013)	HR 1.02 (95% CI 1.02–1.03, *p* < 0.001)
LVEF %	HR 0.93 (95% CI 0.89–0.97, *p* < 0.001)	HR 0.92 (95% CI 0.88–0.96, *p* < 0.001)	HR 0.91 (95% CI 0.86–0.97, *p* = 0.002)	HR 0.90 (95% CI 0.82–0.98, *p* = 0.013)	HR 0.89 (95% CI 0.88–0.94, *p* < 0.001)
LV mass, g/m^2^	NS	HR 1.02 (95% CI 1.00–1.04, *p* = 0.027)	HR 1.03 (95% CI 1.01–1.04, *p* = 0.005)	NS	HR 1.04 (95% CI 1.02–1.05, *p* < 0.001)
LV regional WMA	NS	NS	NS	NS	NS
LV diffuse WMA	HR 10.8 (95% CI 2.26–51.3, *p* = 0.003)	HR 7.62 (95% CI 1.87–30.7, *p* = 0.004)	HR 6.79 (95% CI 1.12–41.3, *p* = 0.037)	NS	HR 22.4 (95% CI 6.41–78, *p* < 0.001)
RVEDVi mL/m^2^	HR 1.03 (95% CI 1.01–1.06, *p* = 0.002)	HR 1.04 (95% CI 1.02–1.07, *p* < 0.001)	HR 1.06 (95% CI 1.03–1.08, *p* < 0.001)	HR 1.07 (95% CI 1.00–1.14, *p* = 0.038)	HR 1.04 (95% CI 1.02–1.06, *p* < 0.001)
RVESVi mL/m^2^	HR 1.04 (95% CI 1.02–1.06, *p* < 0.001)	HR 1.03 (95% CI 1.03–1.07, *p* < 0.001)	HR 1.05 (95% CI 1.03–1.07, *p* < 0.001)	HR 1.10 (95% CI 1.02–1.19, *p* = 0.015)	HR 1.04 (95% CI 1.03–1.06, *p* < 0.001)
RVEF %	HR 0.9 (95%CI 0.86–0.95, *p* < 0.001)	HR 0.90 (95% CI 0.86–0.94, *p* < 0.001)	HR 0.90 (95% CI 0.85–0.95, *p* < 0.001)	HR 0.88 (95% CI 0.80–0.98, *p* = 0.014)	HR 0.91 (95% CI 0.88–0.94, *p* < 0.001)
Edema	NS	NS	NS	NS	NS
Transmural edema	NS	NS	NS	NS	NS
EGE	NS	NS	NS	NS	NS
LGE	NS	NS	NS	NS	NS
LGE > 1 wall	NS	HR 0.21 (95% CI 0.05–0.86, *p* = 0.029)	NS	NS	HR 0.03 (95% CI 0.01–0.13, *p* < 0.001)
Transmural LGE	NS	HR 7.26 (95% CI 1.84–28.7, *p* = 0.005)	NS	NS	NS
LGE mass, g	NS	NS	NS	NS	NS
LGE mass, % of LV	NS	NS	NS	NS	HR 0.73 (95% CI 0.55–0.97, *p* = 0.033)

For abbreviations, see Table 2. NS, non-significant.

## Data Availability

Data will be shared upon reasonable request to the Corresponding Author.
